# Gene Inactivation by CRISPR-Cas9 in *Nicotiana tabacum* BY-2 Suspension Cells

**DOI:** 10.3389/fpls.2016.00040

**Published:** 2016-02-01

**Authors:** Sébastien Mercx, Jérémie Tollet, Bertrand Magy, Catherine Navarre, Marc Boutry

**Affiliations:** Institut des Sciences de la Vie, Université Catholique de LouvainLouvain-la-Neuve, Belgium

**Keywords:** CRISPR, Cas9, plants, suspension cells, gene inactivation, gene targeting

## Abstract

Plant suspension cells are interesting hosts for the heterologous production of pharmacological proteins such as antibodies. They have the advantage to facilitate the containment and the application of good manufacturing practices. Furthermore, antibodies can be secreted to the extracellular medium, which makes the purification steps much simpler. However, improvements are still to be made regarding the quality and the production yield. For instance, the inactivation of proteases and the humanization of glycosylation are both important targets which require either gene silencing or gene inactivation. To this purpose, CRISPR-Cas9 is a very promising technique which has been used recently in a series of plant species, but not yet in plant suspension cells. Here, we sought to use the CRISPR-Cas9 system for gene inactivation in *Nicotiana tabacum* BY-2 suspension cells. We transformed a transgenic line expressing a red fluorescent protein (mCherry) with a binary vector containing genes coding for Cas9 and three guide RNAs targeting *mCherry* restriction sites, as well as a bialaphos-resistant (*bar*) gene for selection. To demonstrate gene inactivation in the transgenic lines, the *mCherry* gene was PCR-amplified and analyzed by electrophoresis. Seven out of 20 transformants displayed a shortened fragment, indicating that a deletion occurred between two target sites. We also analyzed the transformants by restriction fragment length polymorphism and observed that the three targeted restriction sites were hit. DNA sequencing of the PCR fragments confirmed either deletion between two target sites or single nucleotide deletion. We therefore conclude that CRISPR-Cas9 can be used in *N. tabacum* BY2 cells.

## Introduction

*Nicotiana tabacum* cv. Bright yellow 2 (BY-2) suspension cells have been used as a model in a wide range of cellular and physiological studies such as the cell cycle regulation or the effects of environmental conditions and stresses on the plant physiology (Koukalova et al., 1989; Shaul et al., 1996; Fiserova et al., 2006). Moreover BY-2 cells have been shown to be able to produce recombinant proteins, and thus represent an alternative host in the molecular farming field (De Muynck et al., 2010; Schillberg et al., 2013). Typically, cell cultures are grown in contained bioreactors and thus have the advantage of animal and microbial cultures regarding the process control. Recombinant proteins, such as antibodies, can be secreted to the extracellular medium allowing for a purification step much simpler than if they were retained into the cell. The downstream processing costs are thus lower compared to the whole plant system. However, improvements still have to be made regarding the quality and the production rate. For instance, the humanization of glycosylation is required for glycosylated proteins used in therapy. Inactivation of extracellular proteases might be crucial to avoid degradation of recombinant proteins. The possibility of modifying the expression of genes is also an important tool for more basic projects, e.g., aimed at deciphering molecular aspects of a plant cell. These applied or basic targets can be best achieved by genetic engineering tools resulting in either gene silencing or gene inactivation.

Gene silencing by RNA interference has been largely used in plants as well as in plant cells. This approach suffers from the fact that gene silencing is rarely complete and might not be stable over time. From this point of view, gene inactivation by mutation or deletion is more effective but, except for the collections of knock-out lines in *Arabidopsis*, has rarely been implemented in plants because there was no simple method available. Recently, the CRISPR (clustered regularly interspaced short palindromic repeat)/Cas9 (CRISPR-associated) system has been successfully used in a wide range of plant species (Bortesi and Fischer, 2015) but not in plant suspension cells. The system is based on a short RNA guide (sgRNA) which associates to the Cas9 endonuclease to create a double-stranded break (DSB) in the target genomic DNA. As a consequence, mutations are generated through either error-prone non-homologous end-joining (NHEJ) or homology-directed repair (HDR) of the intended cleavage site. NHEJ has been used to generate mutagenic insertions/deletions often leading to gene inactivation. In this report, we tested the CRISPR/Cas9 system in *N. tabacum* BY-2 cells. As a proof of concept we demonstrate the feasibility of using this system to inactivate a reporter gene (*mCherry*) that had been introduced in the genome.

## Materials and Methods

### Plant Cell Cultures

*Nicotiana tabacum* cv. BY-2 (Nagata et al., 1992) suspension cells were grown in the dark at 25°C with agitation on a rotary shaker (90 rpm) in liquid MS medium [4.4 g/L Murashige and Skoog salts (MP BIOMEDICALS, Solon, OH), 30 g/L sucrose, 0.2 g/L KH_2_PO_4_, 2.5 mg/L thiamine, 50 mg/ml myo-inositol, and 0.2 mg/L 2,4-D, pH 5.8 (KOH)].

Cultures were grown in 50 mL of medium in a 250-mL Erlenmeyer flask and a 5% inoculum was transferred each week into fresh medium. Transformed cells were grown on solid medium supplemented with 20 μg/mL of bialaphos.

### Generation of the SC6 Transgenic Line

**FIGURE 1:**
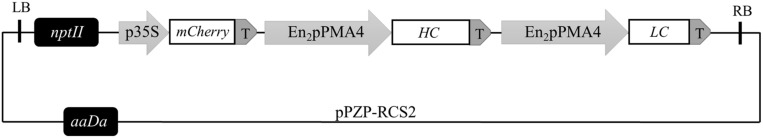
**mCherry binary vector used for transformation of *Nicotiana tabacum* BY-2 cells mediated by *Agrobacterium tumefaciens***. T-DNA left and right borders are indicated by LB and RB, respectively; *nptII*, neomycin phosphotransferase II expression cassette; P35S, double-enhanced cauliflower mosaic virus 35S promoter; *mCherry*, coding sequence for the monomeric fluorescent protein mCherry; En_2_pPMA4, *N. plumbaginifolia* promoter (NpPMA4) reinforced by two copies of the CaMV 35S enhancer; *HC*, heavy chain; *LC*, light chain; T, nopaline synthase polyadenylation sequence (tNOS); aaDa, resistance gene to the aminoglycosides spectinomycin and streptomycin.

A cDNA coding for the monomeric fluorescent protein *mCherry* gene (Shaner et al., 2004) controlled by the double-enhanced cauliflower mosaic virus 35S promoter was inserted in the pPZP-RCS2-nptII-HIgG2-LoBM2 vector (Magy et al., 2014) which contains an expression cassette for the monoclonal antibody Lo-BM2 (**Figure 1**). The binary vector was transferred into *Agrobacterium tumefaciens* LBA4404virG (van der Fits et al., 2000) by electroporation. *A. tumefaciens* was cultured for 16 h in 50 ml of culture medium supplemented with antibiotics (20 μg/ml rifampicin, 40 μg/ml gentamicin, 50 μg/ml kanamycin) as well as 100 μM acetosyringone. After harvesting by centrifugation at 3,500 *g*, the bacteria were resuspended at an O.D. of 1 (600 nm) in MS medium supplemented with 100 μM acetosyringone and 10 mM MgSO_4_. The suspension was incubated for 3 h at room temperature with shaking and then 40 ml were mixed with 4 ml of a 6 days old BY-2 cell culture and poured onto solid MS medium containing 100 μM acetosyringone. After 2 days at 25°C, the cells were washed and cultured in liquid MS medium for 3 days and plated on solid MS media supplemented with 500 μg/ml cefotaxim, 400 μg/ml carbenicillin, 100 μg/ml kanamycin. A line displaying mCherry fluorescence (SC6) was chosen as a target for further work.

### Cas9 and sgRNA Plasmid Construction and Plant Cell Transformation

pFGC-pcoCas9 was a gift from Jen Sheen (Addgene plasmid # 52256). The three sgRNAs were constructed by overlapping PCR, inserted into the pGEM-T-easy vector (Promega), sequenced, and then introduced into either the AscI (sgRNA1), PacI (sgRNA2), or SbfI (sgRNA3) cloning sites of the pFGC-pcoCas9 binary vector (Li et al., 2013). The vector was transferred into *A. tumefaciens* LBA4404virG (van der Fits et al., 2000) by electroporation. Transformation of *N. tabacum* BY-2 cells was performed as indicated above except that the selection was on 20 μg/ml bialaphos.

### RNA Extraction and RT-PCR

BY-2 cells (100 mg) were collected 3 days after co-cultivation with *A. tumefaciens*, frozen in liquid nitrogen, and ground in powder. Then, RNA was extracted with the Spectrum^TM^ Plant Total RNA Kit (Sigma–Aldrich). cDNA was synthesized by mixing 0.2 μg RNA, 10 μM oligo dT, 10 μM oligo gRNA-mCherry-RT-R (hybridizing to sgRNAs), and H_2_O up to 15 μl. After incubation for 5 min at 70°C, the sample were placed on ice and 5 μl 5x M-MLV buffer (Promega), 1.25 μl dNTPs (10 mM), 25 U RNase inhibitor, 200 UM-MLVRT (Promega), and 16.75 μl H_2_0 were added. The sample was incubated for 1 h at 42°C. PCR was performed according to the GoTaq^®^ DNA Polymerase protocol (Promega).

### Analysis of Genome Modification

Genomic DNA was extracted from stable transgenic transformants from the bialaphos selection and SC6 non-transformed cells. PCR was performed using primers flanking *mCherry* (**Table 1**) and the amplified fragments were electrophoresed on an ethidium bromide-stained agarose gel (2%). For the restriction fragment length polymorphism (RFLP) analysis, the PCR product was digested with the corresponding enzymes chosen for each target site and electrophoresed on an ethidium bromide-stained agarose gel (2%). For further characterization the bands were purified and sequenced.

**Table 1:** Primers used in this study.

Primer name	Sequence (5′ to 3′)	Usage
U6-AscI-F	CGAGGCGCGCCAGAAATCTCAAAATTCCG	For creating
gRNA-mCherry1-R	TGTGCACCTTGAAGCGCATGCAATCACTACTTCGTCTCT	sgRNA1
gRNA-mCherry1-F	GCATGCGCTTCAAGGTGCACAGTTTTAGAGCTAGAAATAGC	
gRNA-AscI-R	CGAGGCGCGCCTAATGCCAACTTTGTACA	

U6-PacI-F	CGATTAATTAAAGAAATCTCAAAATTCCG	For creating
gRNA-mCherry2-R	GGGACATCCTGTCCCCTCAGCAATCACTACTTCGTCTCT	>sgRNA2
gRNA-mCherry2-F	GCTGAGGGGACAGGATGTCCCGTTTTAGAGCTAGAAATAGC	
gRNA-PacI-R	CGATTAATTAATAATGCCAACTTTGTACA	

U6-SbfI-F	CGACCTGCAGGAGAAATCTCAAAATTCCGA	For creating
gRNA-mCherry3-R	ACGTGAAGCACCCCGCCGACAATCACTACTTCGTCTCT	sgRNA3
gRNA-mCherry3-F	GTCGGCGGGGTGCTTCACGTGTTTTAGAGCTAGAAATAGC	
gRNA-SbfI-R	CGACCTGCAGGTAATGCCAACTTTGTACA	

Cas9RT-F	CTACGTTGGACCACTTGC	RT-PCR
Cas9RT-R	ACAGAATCGAAGCACTCG	
gRNA-mCherry1RT-F	CTTCAAGGTGCACAGTTTT	
gRNA-mCherry3RT-F	GGGTGCTTCACGTGTTT	
gRNA-mCherryRT-R	ACTCGGTGCCACTTTTTC	

mCherry-F	AAGGGCGAGGAGGATAAC	PCR
mCherry Rev1	CAGCCTCTGCTTGATCTC	

## Results

### Design of the CRISPR/Cas9 System Targeting mCherry in Nicotiana tabacum BY-2 Cells

**FIGURE 2:**
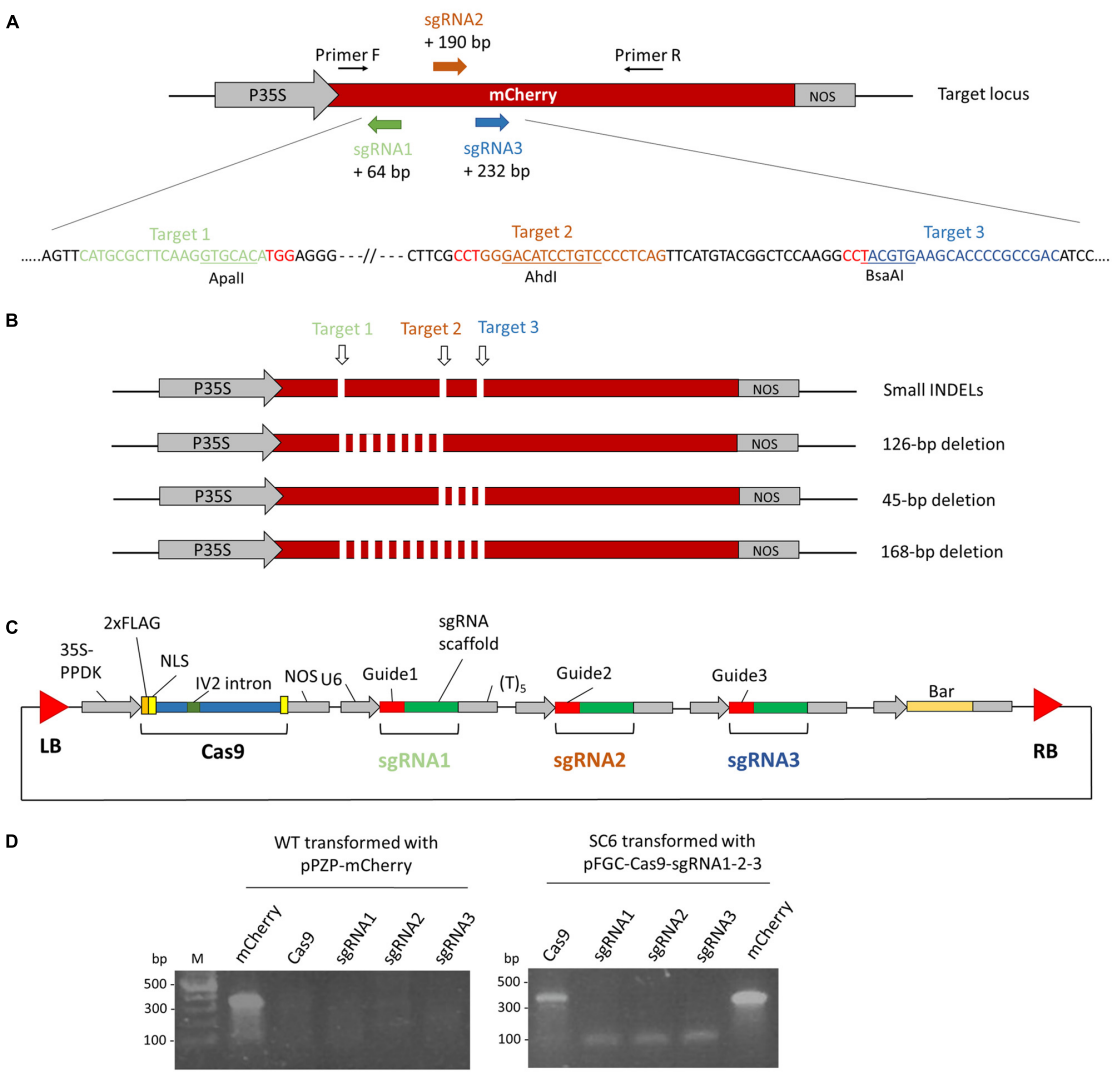
**Genome editing in *Nicotiana tabacum* BY-2 cells using a transgenic line expressing a fluorescent protein mCherry (SC6) by CRISPR/Cas9. (A)** Schematic representation of the *mCherry* gene and the three target sites. Three regions of *mCherry* were selected for the presence of a restriction site (ApaLI, BsaAI, and AhdI) to facilitate the mutation analysis by RFLP. Primers F and R were used to amplify *mCherry* in order to examine the mutations. The distance between the three target sites and the mCherry translation start is indicated. P35S: CaMV 35S transcription promoter. NOS, nopaline synthase terminator. The sequence corresponding to the sgRNA is displayed below in colors. The PAM sequences are in red. **(B)** Schematic representation of the expected mutation types. INDELs can be created by a single break. Breaks occurring at two sites simultaneously result in a deletion between those sites. **(C)** Schematic representation of the pFGC-Cas9-sgRNA1-2-3 vector used in this study. Cas9 is controlled by the 35S-PPDK transcription promoter and three sgRNAs were integrated in the vector downstream of the U6 transcription promoter. A *bar* gene permits the selection of transformants. **(D)** Expression analysis by RT-PCR of *Cas9* and the sgRNAs 3 days post-transformation with *A. tumefaciens* carrying the pFGC-Cas9-sgRNA1-2-3 vector. As a control, WT BY-2 cells were transformed with a vector (pPZP-mCherry) expressing mCherry.

To test the potential of CRISPR/Cas9 to generate a gene knockout in *N. tabacum* BY-2 cells we obtained a transgenic line (SC6) containing a reporter gene expressing the mCherry fluorescent protein. This line was obtained after transformation of BY-2 cells with a vector previously used to express an antibody (Magy et al., 2014) in which we inserted the *mCherry* gene (**Figure 1**). A transgenic line, SC6, was chosen for its high mCherry expression. Three regions of *mCherry* were targeted by three sgRNAs (**Figure 2A**). We selected the target sites for the presence of a restriction site to facilitate the identification of mutations by an RFLP assay. We expected short INDELs at different target sites but also deletions between two target sites if a break occurs at two sites simultaneously (**Figure 2B**). We constructed the pFGC-Cas9-sgRNA1-2-3 binary vector containing an *Arabidopsis* codon-optimized version of Cas9 controlled by the 35S-PPDK promoter, a *bar* gene for selection, and three sgRNAs (controlled by the U6 promoter) targeting *mCherry* (**Figure 2C**). The sgRNAs of each target site were generated by overlapping PCR and cloned into the pFGC-Cas9 vector. The SC6 cell line was transformed with *A. tumefaciens* carrying pFGC-Cas9-sgRNA1-2-3. Three days after transformation, we sought to determine whether the genes for Cas9 and the sgRNAs were expressed. At that stage, no selection had been applied and transient expression was checked in the whole cell suspension by RT-PCR analysis. Transcripts for both *cas9* and sgRNA were identified (**Figure 2D**). Afterward, the cell suspension was spread on a selection medium containing bialaphos to isolate transformed calli.

### CRISPR/Cas9 Induces INDELs and Fragment Deletion in *mCherry*

**FIGURE 3:**
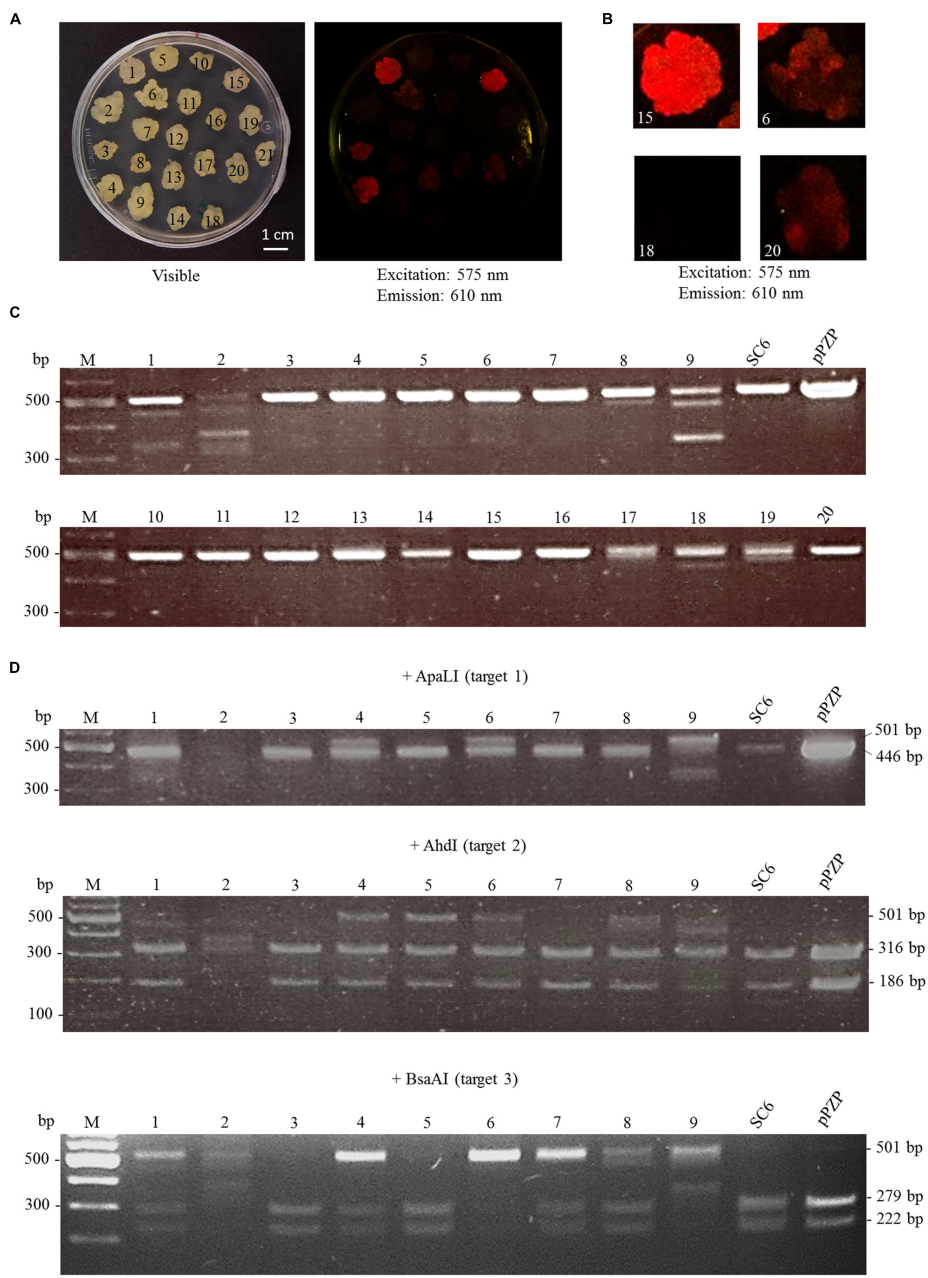
**Analysis of genome editing at the *mCherry* locus**. Cells of the SC6 line were transformed with pFGC-Cas9-sgRNA1-2-3 targeting *mCherry*. Transformants appeared after 3 weeks on bialaphos selection medium and were transferred twice on new selection medium. **(A)** Picture of the calli under visible light (left) and fluorescence of mCherry (right). **(B)** Close-up of four calli: lines 15 (homogenous mCherry fluorescence), 18 (no mCherry fluorescence) 6 and 20 (heterogenous mCherry fluorescence). **(C)** Genome editing in transformed calli was monitored by PCR amplification of *mCherry.* Deletion between two target sites occurred in seven out of 20 transformants (lines 1, 2, 8, 9, 17, 18, 19). pPZP: amplification of *mCherry* directly from the plasmid pPZP-mCherry. **(D)** Genotyping of nine transformants by RFLP analysis. The PCR fragments from lines 1 to 9 displayed in **(C)** were subjected to digestion by the indicated restriction enzymes.

**FIGURE 4:**
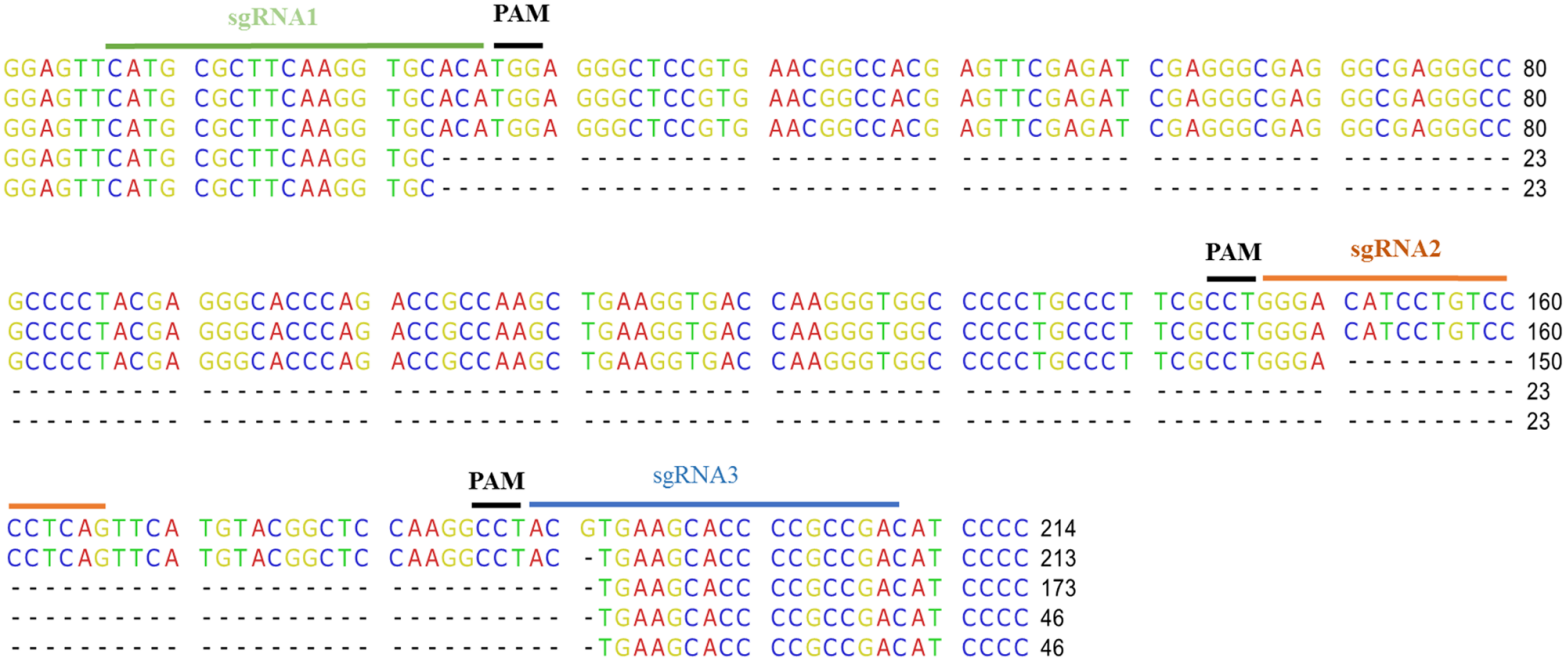
**Sequence of the edited sites**. PCR fragments amplified from the *mCherry* genes were cloned and sequenced. The sequence of four clones is displayed below the sequence of the non-edited mCherry. The sgRNA and the PAM positions are indicated.

Random transformants were transferred twice on a fresh bialaphos selection medium and then checked for the fluorescence of mCherry. Loss of fluorescence was observed in 19 out of 21 transformants. This loss was partial (chimeras) for 15 lines and complete for the other four (**Figures 3A,B**). To confirm that the loss of fluorescence was due to mutations in *mCherry*, genomic DNA extraction was performed followed by PCR amplification of the target region (**Figure 3C**). Seven out of 20 lines tested showed, in addition to the undeleted fragment, shortened fragments, which correspond to deletions between two target sites. To determine whether INDELs occurred within the full size fragments, RFLP analysis was performed directly on the PCR products of the first nine clones (**Figure 3D**). Three out of nine lines exhibited a band partially resistant to ApaLI digestion, indicating the creation of short INDELs in target 1. Five out of the nine lines were mutated in target 2, and five in target 3. CRISPR-Cas9 together with the three sgRNAs thus created mutations in all the lines except for line 15, in which a loss of fluorescence was not observed. However, the lines were not homogenous for the mutations. Instead, for most of them, a mix of INDELs at two or three targets was identified. To further characterize mutations, four PCR fragments (three with a shorter size and one with a full size) were cloned and sequenced (**Figure 4**). The full size fragment displayed a single base deletion in target 3. One shortened fragment resulted from a deletion between targets 2 and 3, and the last two fragments, from a deletion between targets 1 and 3.

## Discussion

In the present study, we showed that CRISPR/Cas9 can be used as a powerful tool for engineering the *N. tabacum* BY-2 genome. Previous studies showed the possibility to induce mutations in the genome of various plants or plant protoplasts (Jiang et al., 2013; Li et al., 2013; Gao et al., 2015), but plant suspension cells had not yet been used as a target.

Since the efficiency of the CRISPR/Cas9 in *N. tabacum* BY-2 cells was unknown, we targeted the marker gene *mCherry*, the expression of which could be monitored by fluorescence. However, selection of non-fluorescent transformants was not necessary as among the 21 transformants randomly chosen, four had lost their mCherry fluorescence. However, 15 chimeric calli were also observed. This indicates that Cas9 had not yet provoked any genomic break in some of the cells. An explanation is that the initial transformant was actually a mix of two transformation events, one of which did not lead to the expression of Cas9 and/or sgRNAs, either because of incomplete transfer of the T-DNA, or because of a position effect. Another explanation is that after transformation, a first target site was hit after the first cell division, resulting in a chimeric callus.

On the whole, RFLP analysis indicated that all three *mCherry* targets were hit. In addition, the deletion of a fragment between two targets was also observed, which shows that in some cases, Cas9 had hit two targets before non-homologous end-joining reparation had occurred at each site. This observation suggests that homology-directed repair might be feasible in suspension cells. When examining individual transformants, heterogeneity was usually observed, with a mix of hits at one, two, or three targets. However, no case was found where the three sites were homogenously mutated, and only one case out of nine was found where a single site was homogenously mutated (line 6 at target 3). As hypothesized before, this suggests that after transformation with *A. tumefaciens* cell division of the transformed cells usually occurred before a target was mutated by Cas9. In this case, mutations appear over time when the callus grows, resulting in chimeric clones. However, homogenous mutant lines can be obtained by subcloning. When too diluted, plant suspension cells do not grow, thus preventing isolated colonies from being obtained. However, an alternative consists of mixing transformed cells with an excess of feeding wild-type cells and selecting isolated calli on a selection medium (Kirchhoff et al., 2012).

## Conclusion

Targeting three sites of a gene of interest, we showed that it is possible to use CRISPR/Cas9 to knock-out this gene. Besides single mutations at one of the targets, double breaks and deletion of the intervening sequence were also observed, opening the way to homologous recombination by homology-directed repair.

## Author Contributions

SM performed most of the research, analyzed the data and wrote the manuscript. JT and BM obtained the SC6 transgenic line. CN supervised the research and wrote the manuscript. MB conceived and designed the project and wrote the manuscript.

## Conflict of Interest Statement

The authors declare that the research was conducted in the absence of any commercial or financial relationships that could be construed as a potential conflict of interest.
